# The effect of transcranial direct current stimulation: a role for cortical excitation/inhibition balance?

**DOI:** 10.3389/fnhum.2013.00602

**Published:** 2013-09-24

**Authors:** Beatrix Krause, Javier Márquez-Ruiz, Roi Cohen Kadosh

**Affiliations:** ^1^Department of Experimental Psychology, University of OxfordOxford, Oxfordshire, UK; ^2^Division of Neurosciences, Pablo de Olavide UniversitySeville, Spain

**Keywords:** transcranial direct current stimulation (tDCS), excitation, inhibition, GABA, glutamate, cognition

## Abstract

Transcranial direct current stimulation (tDCS) is a promising tool for cognitive enhancement and neurorehabilitation in clinical disorders in both cognitive and clinical domains (e.g., chronic pain, tinnitus). Here we suggest the potential role of tDCS in modulating cortical excitation/inhibition (E/I) balance and thereby inducing improvements. We suggest that part of the mechanism of action of tDCS can be explained by non-invasive modulations of the E/I balance.

## Introduction

Cognitive enhancement is a popular topic in the neuroscience community. Non-invasive neuromodulation methods, such as transcranial direct current stimulation (tDCS) can either increase (e.g., anodal) or decrease (e.g., cathodal) cortical excitability (Nitsche and Paulus, 2001; Nitsche et al., 2003) and thereby modulate cortical activity levels.

At a cellular level, the applied external electric field modifies the transmembrane potential differences by forcing the displacement of intracellular ions which cancel the generated intracellular field and thereby modify the spike firing probability (Bikson et al., 2004; Ruffini et al., 2013). With sufficient tDCS duration, synaptically driven aftereffects are induced (Bindman et al., 1964). The final effects of tDCS depend on the individual neural morphology (Radman et al., 2009), the orientation of somato-dendritic axes, and the neural pathways with respect to the electric field (Bikson et al., 2004; Kabakov et al., 2012).

tDCS has positive effects in a variety of clinical conditions such as Parkinson’s disease, tinnitus, chronic pain, stroke, and even childhood psychosis (e.g., Fregni et al., 2006; Song et al., 2012; David et al., 2013; Khedr et al., 2013; Moreno-Duarte et al., 2013), but also in healthy individuals (Jacobson et al., 2012; Kuo and Nitsche, 2012; Cohen Kadosh, 2013). It is therefore considered a promising neurorehabilitation tool. Moreover, tDCS has recently been suggested as a possible tool to improve learning disabilities in children (Krause and Cohen Kadosh, 2013; Vicario and Nitsche, 2013). A crucial question remains to be answered: how exactly does tDCS modify such diverse conditions in both the typical and atypical brain?

## Neurotransmitters and tDCS

Magnetic resonance spectroscopy (MRS) studies have shown that anodal tDCS reduces local concentrations of the inhibitory neurotransmitter gamma-aminobutyric acid (GABA), whereas cathodal tDCS reduces excitatory glutamate levels (Stagg et al., 2009; Clark et al., 2011).

Others have suggested that local GABA reductions co-occur with learning and performance improvements (Floyer-Lea et al., 2006) and that the magnitude of regional GABAergic changes during anodal tDCS reflects the degree of learning (Stagg et al., 2011). Namely, the further GABA is decreased, the larger the observed learning effect. Such disinhibition may lead to the unmasking of hidden excitatory connections (Jacobs and Donoghue, 1991) and thereby allow for the induction of activity-dependent long-term potentiation (LTP). LTP in turn is capable of inducing cortical reorganization, most likely by increasing local synaptic effectiveness (Hess and Donoghue, 1994), which in turn might alter deficient network processing.

In addition, data coming from animal experiments have demonstrated the implication of N-methyl-D-aspartate (NMDA) receptors and brain-derived neurotrophic factor (BDNF) in the synaptic potentiation of the motor cortex after anodal tDCS (Fritsch et al., 2010). Moreover, local administration of the adenosine A1 receptor antagonist 8-cyclopentyl-1,3-dipropylxanthine (DCPCX) in the somatosensory cortex of alert rabbits prevented long-term depression induced by cathodal tDCS (Marquez-Ruiz et al., 2012). These data suggest that beyond GABA and glutamate, other neurochemicals may be involved in the mechanisms underlying long-term tDCS effects.

## Excitation/Inhibition (E/I) balance

Homeostatic control of cortical excitability and induction of plasticity are crucial for allowing efficient information transfer in the brain, (Turrigiano and Nelson, 2000). This means that while plastic changes occur, the network must still maintain a certain amount of stability in order to produce meaningful output. The dysregulation of cortical excitability may thus lead to symptoms seen in various central nervous system disorders (Eichler and Meier, 2008), depending on the area(s) in which the imbalance occurs. For instance, regional abnormalities in GABA concentrations have been found in neuropsychiatric disorders, such as schizophrenia (Goto et al., 2009; Yoon et al., 2010; Yizhar et al., 2011; Rowland et al., 2013), autism (Kubas et al., 2012; Rojas et al., 2013), insomnia (Morgan et al., 2012), and panic disorder (Long et al., 2013).

However, GABA concentrations alone may not fully explain different kinds of cognitive deficits. For instance, if glutamatergic excitation is increased as well, we would not expect to observe performance abnormalities. Most studies so far have only looked at glutamate and GABA in isolation (e.g., Goto et al., 2009; Yoon et al., 2010; Kubas et al., 2012; Rojas et al., 2013).

We suggest that the regional cortical excitation/inhibition (E/I) balance, measured by ratios of glutamate/GABA, may provide more meaningful interpretations of individual cognitive performance and deficits than glutamate or GABA alone. GABA and glutamate contribute in a complementary fashion to high-level prefrontal cognitive performance in healthy adults (Jocham et al., 2012). Furthermore, individuals with autism or schizophrenia show higher E/I ratios compared to healthy controls (Rubenstein and Merzenich, 2003), and this has been suggested to be related to behavioral and cognitive deficits (Yizhar et al., 2011). Similarly, regional increases in glutamate (Carrey et al., 2007; Arcos-Burgos et al., 2012) and reduced levels of GABA (Edden et al., 2012) have been found in several different brain areas of individuals with Attention-deficit hyperactivity disorder (ADHD). These findings lend support to the view that E/I balance plays a major role in normal cognition, as well as the symptomatic patterns of a variety of clinical conditions.

Using cathodal tDCS to artificially decrease E/I in ADHD for example could be beneficial. Cathodal stimulation may restore the elevated E/I balance towards a more typical level in targeted regions, which require greater baseline inhibition, in order to reduce irrelevant output. For instance, in healthy adults, applying cathodal stimulation to prefrontal regions has been shown to lead to improved attentional processing. This likely enhances prefrontal filtering of irrelevant information (Weiss and Lavidor, 2012).

**Figure 1:**
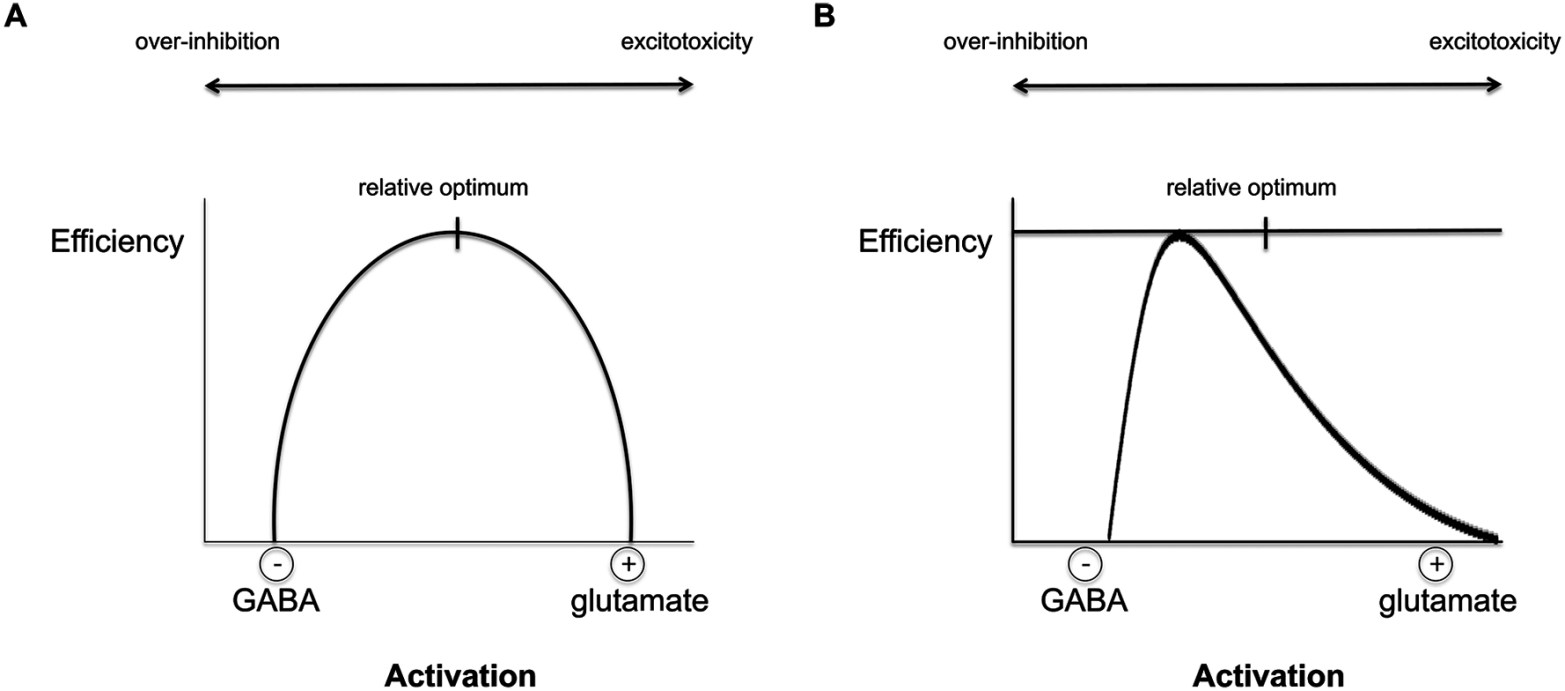
**The relationship between excitation/inhibition (E/I) balance and efficiency of a given cortical region**. **(A)** According to the current hypothesis, E/I balance within a brain area can be viewed as an inverted-U shape in which the optimal performance is achieved when excitation and inhibition interact efficiently, allowing for both plasticity and stability. The degree of baseline E/I might, however, differ per brain region and individual. If the optimal balance is achieved, homeostatic control of activity-dependent plasticity and synaptic efficiency are possible and can lead to meaningful behavioral output. Deviations from the ideal balance are associated with atypical behavior and the severity of the deficit may vary with the degree of imbalance. tDCS can be used to target and restore the individual abnormalities in E/I imbalance in different neurological conditions. Only a moderate level of activation, i.e., balanced E/I levels, can reach the optimal level of processing efficiency and allow for homeostatic plasticity. High levels of GABA can lead to cortical over-inhibition that will reduce network output, whereas hyperactive glutamatergic activity can lead to excessive output and eventually to excitotoxicity and cell death (Faden et al., 1989; Belousov, 2012). **(B)** An example of the distribution of E/I balance in the healthy population: the finding that most anodal tDCS studies report behavioral improvements suggests that the distribution may be skewed with the majority showing non-optimal E/I ratio. However, for some individuals with increased E/I ratios, anodal tDCS will shift the non-pathological imbalance even further towards over-activation and therefore reduce behavioral outcomes.

The direction of the E/I imbalance may determine the behavioral outcome depending on the particular brain area and appears to be different in different clinical populations. Therefore, a fundamental understanding of individual differences in E/I ratio would allow for optimization of the choice of tDCS parameters for each individual in terms of polarity, intensity, duration, etc. (Figure 1).

## Discussion

This simple, but elegant model explains individual differences in cognitive performance and cognitive deficits, as well as the polarity-specific effects of tDCS on cognition, and can be extended to non-cognitive domains, as well (e.g., pain: Harris and Clauw, 2012). Nevertheless, the effects of tDCS on neural network dynamics, more specifically at neurotransmitters concentrations, are largely unknown.

At a microscopic level, glutamate is released by pyramidal cell synapses and thalamic synaptic inputs, whereas GABA is mainly released by a variety of interneurons (Nicoll et al., 1990; McCormick, 1992). Animal experiments using brain slices suggest that pyramidal cells in layer V are the most sensitive to the effects of weak electric fields applied over the skull surface (Radman et al., 2009). Thus, anodal and cathodal tDCS are expected to increase or decrease, respectively, the membrane potential of pyramidal cells and thereby alter the glutamatergic tone in the cortex.

Nevertheless, glutamate levels not only depend on pyramidal cells but also on input from thalamic projections. It has been recently shown in both humans (Polania et al., 2012) and alert rabbits (Marquez-Ruiz et al., 2012) that tDCS also modifies thalamocortical synapses by means of glutamate release from sensory afferents. As pyramidal cells project to different types of interneurons, it is expected that the modulation of glutamate levels correlates with GABA release. However, a recent computational modeling study based on in-vivo experimental data proposed that tDCS may induce opposing effects on different types of interneurons (Molaee-Ardekani et al., 2013), suggesting a more complex scenario. Finally, in order to fully understand the mechanism underlying E/I balance, other factors, such as levels of BDNF or cortical adenosine and cortical oscillations must also be taken into consideration. For example, it has been shown in brain slices that weak direct current (DC) stimulation may modulate slow-wave (Frohlich and McCormick, 2010) and gamma oscillations (Reato et al., 2010) related with E/I balance in the cortex (Shu et al., 2003; Haider et al., 2006; Atallah and Scanziani, 2009).

According to the current evidence, tDCS is likely to reinstate an optimal E/I balance that allows for optimal homeostatic plasticity in learning and cognition, if applied adequately to each individual’s predispositions. If this consistently proves to be the case, a variety of cortex-based clinical conditions including atypical brain development may be successfully treated using tDCS. So far, there is little research investigating the relationship between E/I balance and cognition. The assessment of this balance in different clinical, neurological and neuro-developmental disorders will help refine tDCS strategies for treatment in the future. Whether electrical stimulation can also modulate E/I balance in the case of transcranial random noise stimulation (tRNS) and transcranial alternating current stimulation (tACS) is currently unknown and requires further exploration.

## Conflict of interest

Roi Cohen Kadosh filed a patent for an apparatus for improving and/or maintaining numerical ability.
